# A 3D Brillouin microscopy dataset of the *in-vivo* zebrafish eye

**DOI:** 10.1016/j.dib.2020.105427

**Published:** 2020-03-17

**Authors:** Héctor Sánchez-Iranzo, Carlo Bevilacqua, Alba Diz-Muñoz, Robert Prevedel

**Affiliations:** a Cell Biology and Biophysics Unit, European Molecular Biology Laboratory, Heidelberg, Germany; b Developmental Biology Unit, European Molecular Biology Laboratory, Heidelberg, Germany; c Epigenetics and Neurobiology Unit, European Molecular Biology Laboratory, Monterotondo, Italy; d Molecular Medicine Partnership Unit, European Molecular Biology Laboratory, Heidelberg, Germany

**Keywords:** Brillouin microscopy, Zebrafish larvae, Brillouin scattering, Mechanobiology, Elasticity, Viscosity

## Abstract

In this work we present three-dimensional (3D) measurements of Brillouin scattering spectra of the *in-vivo* zebrafish larvae eye. This dataset was obtained by Brillouin microscopy, an emerging all-optical and non-contact technique that gives access to material properties through the process of Brillouin scattering. Herein, we share a representative 3D dataset of spectral properties of 48–52 h post-fertilization (hpf) zebrafish embryos. These spectral properties can be related to a complex longitudinal modulus and thus elastic and viscous properties given knowledge of refractive index and material density. The dataset encompasses the crystalline lens as well as several different retinal layers. This data provides a valuable resource as well as a starting point for researchers interested in the mechanobiology of vertebrate eye development.

Specifications table

**Table:** 

Subject	Biophysics
Specific subject area	Brillouin microscopy
Type of data	Image
	Figure
How data were acquired	Confocal Brillouin microscope, consisting of a self-built, dual-stage VIPA spectrometer coupled to an inverted microscope (Zeiss Axiovert 200 M). Self-written LabView and Matlab software to collect and analyse spectral data, respectively.
Data format	Raw
	Analyzed
	Filtered
Parameters for data collection	Zebrafish larvae at 48–52 hpf were mounted in 0.6% agarose. Right eye was imaged.
Description of data collection	Brillouin spectra were obtained over a region of 274 × 200 × 40 µm, with a step size of 2 × 2 × 20 µm in lateral (x-y) and axial (z) directions, respectively, with a collection time of 180 ms per pixel. Illumination wavelength and power were 532 nm and <10 mW, respectively.
Data source location	Institution: European Molecular Biology Laboratory
	City/Town/Region: Heidelberg
	Country: Germany
Data accessibility	Repository name: Mendeley Data
	Data identification number: 10.17632/n9mzxxmhdm.3
	Direct URL to data: https://data.mendeley.com/datasets/n9mzxxmhdm/3

## Value of the data

• These data and the associated analysis pipeline provide a walkthrough on how to obtain spectral information from Brillouin scattering experiments. These can be related to a complex longitudinal modulus and thus elastic and viscous properties given spatial knowledge of refractive index and material density.
• Our representative dataset, which is related to the elasticity and viscosity of the zebrafish eye at high-frequency, provides a first glimpse into mechanical differences of neuronal layers in a vertebrate retina.
• These data will be of interest to all scientists who are interested in the mechanobiology of tissue morphogenesis. In particular, the vertebrate eye contains several neuronal layers whose mechanical differences can be resolved by Brillouin microscopy. The analysis of their development when done over time by Brillouin microscopy might directly inform our understanding of the role of mechanics in the morphogenesis of this complex organ.
• This is a rich dataset for the Brillouin microscopy community as the zebrafish eye contains several cell layers with different cell density and fate in close spatial proximity. Their analysis could thus contribute to our understanding of the biological origin of the Brillouin signal.
• The data can be further used to test and develop alternative or more sophisticated analysis pipelines and algorithms to extract visco-elastic information from Brillouin spectra of biological samples.

## 1 Data description

**Fig. 1:**
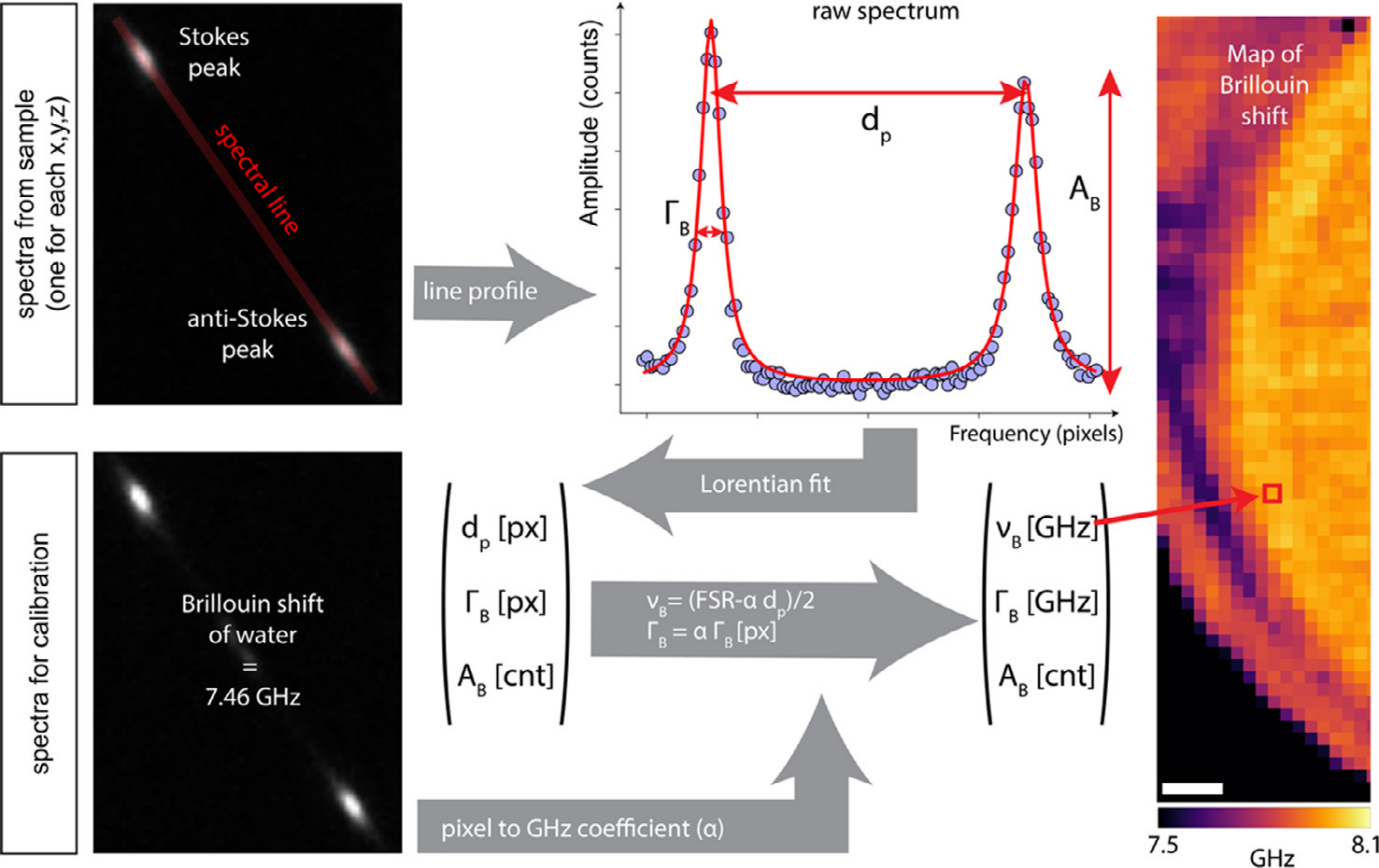
Workflow of data analysis. An image from the EM-CCD is acquired for each point in the sample; the raw spectrum is determined by taking a line profile from the spectral line. A Lorentzian fit allows to retrieve the distance between the peaks (d_p_), the width (Γ_B_ - FWHM) and the amplitude (A^B^) of the peaks. A pixel to frequency (GHz) conversion coefficient is determined from a Brillouin spectrum acquired from water (7.46 GHz) and used to convert the raw values (in pixels) given by the fit to frequency (GHz). By repeating the same analysis for each confocal volume (pixel) in the sample, a Brillouin maps can be reconstructed (scale bar 10 µm).

The shared raw data are individual camera frames, one collected for each spatial (x,y,z) position in the sample (Fig. 1). The camera records the one-dimensional frequency spectrum of the elastically scattered (‘Brillouin’) light, and contains the so-called Stokes and Anti-Stokes Brillouin peaks over a range of ±15 GHz frequency shift. Note that the Rayleigh light, denoting the in-elastically scattered light at zero frequency shift, is blocked by a physical mask to avoid saturation of the camera. During the analysis, the spectrum is fitted by a (double-)Lorentzian function, and the fit parameters (peak location, width and amplitude) are converted from pixels to frequency (GHz) using a previously established calibration routine (see Fig. 1 and Methods). These extracted spectral parameters (frequency shift, spectrum width and amplitude) then constitute the analysed data. The raw data can be accessed here: https://data.mendeley.com/datasets/n9mzxxmhdm/3.

**Fig. 2:**
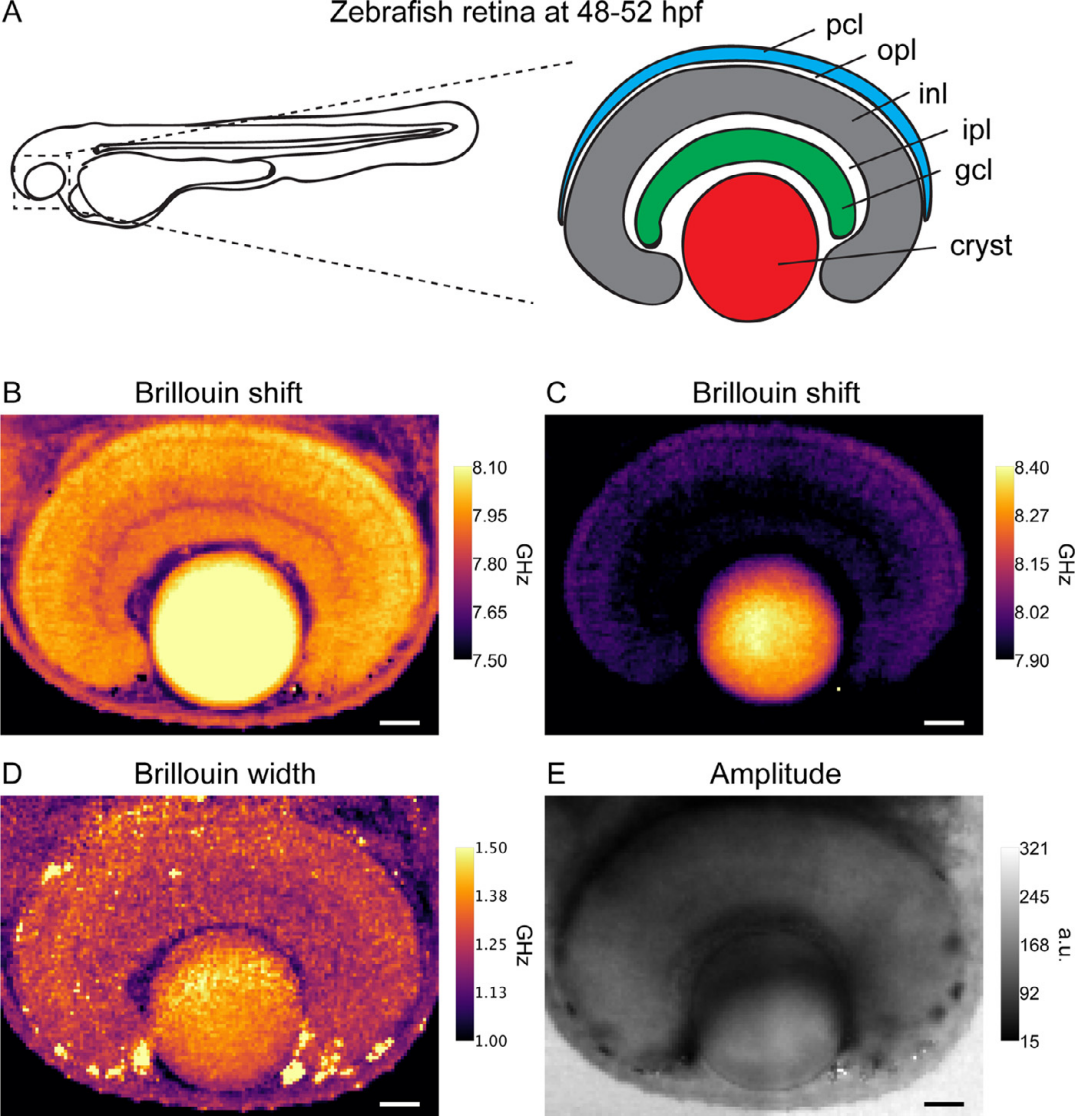
(A) Schematic representation of a zebrafish eye at 48–52 hpf. pcl, photoreceptor cell layer; opl, outer plexiform layer; inl, inner nuclear layer; ipl, inner plexiform layer; gcl, ganglion cell layer; cryst, crystallin. (B, C) Brillouin shift of the right eye of a zebrafish at 48–52 hpf and at ∼50 µm depth. mpl-inferno lookup table was used as a color scale. Two different scales were used to allow for better visualization of retinal layers (B) or the crystallin (C). (D) Brillouin width (FWHM) of the Anti-Stokes peak. (E) Scattering amplitude (average of Stokes and Anti-Stokes peaks). Scale bars 25 µm.

Fig. 2 shows a representative example (from 3 biological replicates) of our experimental results, displaying a single 2D (x,y) plane of our 3D dataset of the *in-vivo* 48–52 hpf zebrafish eye. These filtered data show the spatial distribution of frequency shift υ_B_ (a proxy for ‘elasticity’, Fig. 2C), spectral width Γ_B_ (related to ‘viscosity’, Fig. 2D) and signal amplitude A_B_ (a relative measure of scattering cross-section and absorption, Fig. 2E), and could thus give insight into the mechanobiology of this vertebrate organ, in particular in the spatial heterogeneity of mechanical properties and their potential origin from different neuronal types and cell densities [1].

## 2 Experimental design, materials, and methods

### 2.1 Brillouin microscopy

The Brillouin microscope is composed of a commercial inverted microscope body (Zeiss Axiovert 200M) and a self-built spectrometer employing two virtually-imaged phase arrays (VIPA) which separate the Brillouin scattered light from the inelastic background (Rayleigh). The illumination light source is a single-frequency mode, 532-nm continuous-wave laser (Torus, Laser Quantum) and is focused by a Zeiss Plan-Apochromat 40x, 1.0 NA objective. Sample positioning is achieved with a Piezo translation stage (P-545.3R8H, Physik Instrumente). The Brillouin scattered light is collected in so-called backscattering direction by the same objective and fiber-coupled to ensure confocality and to send the light to the spectrometer for analysis. A quarter-wave plate (QWP) in combination with a PBS separates the illumination from the backscattered light. The scheme conceptually follows [2] and a more detailed description of the experimental apparatus employed here can be found in [3]. In brief, our microscope has a mechanical lateral (axial) resolution of 0.3(1.6)µm as well as a spectrometer resolution of 460 MHz.

### 2.2 Zebrafish imaging

In this work, we have used 48–52 hpf zebrafish. Phenylthiourea was added at 0.003% since 10 hpf to prevent pigmentation. At the time of acquisition, fish were embedded in 0.6% low gelling temperature agarose (A0701, Sigma) and anesthesized with 0.016% tricaine (protocol adapted from Ch. 4.3 in Ref. [4]). The right eye of the fish was imaged. After the data acquisition, the larvae were released from the agarose and behaved, as well as developed, normally after cessation of sedation. Brillouin spectra were obtained over a region of 274 × 200 × 40 µm starting at ∼30 µm depth, with a step size of 2 × 2 × 20 µm in lateral (*x*-*y*) and axial (*z*) directions, respectively, with a dwell time of 180 ms per pixel. Illumination power was <10 mW. Under these conditions, the Brillouin shift (linewidth) measurements had a precision of ∼10 (18) MHz. These parameters were carefully chosen to optimize the measurement precision while keeping imaging time and photodamage risk minimal.

### 2.3 Data analysis

The dataset is organized in two folders, which contain the data for two 3D regions of the eye. Each folder hosts the unprocessed camera frames for each spatial position (named spectrum[*index*]x[*um*]y[*um*]z[*um*]), as well as reference spectra of water (named `reference-[*index*]'). Raw data (camera frames) were analyzed in real-time with a self-written Labview program, following the same workflow as the accompanying Matlab script (`BrillouinDataAnalysis'), which can be summarized as follows (also see screenshots accompanying the analysis script) [3]: First, the spectral region-of-interest, the spectral axis and the rough position of the Brillouin peaks and background are manually selected through a graphical user interface. Next, Lorentzian functions are fitted to the extracted pixels in order to obtain the position, width and intensity of the Brillouin peaks (see Fig. 1). In order to calibrate the frequency axis, a reference measurement of a water sample is acquired after every 50 measurements (∼10 s). The conversion between camera pixels and GHz is performed assuming a linear relationship between the distance of the Brillouin peaks on the camera (*d_p_*) and the corresponding Brillouin shift $\left(\upsilon_{B}\right):\upsilon_{B}=\left(FSR-\alpha \cdot d_{p}\right)/2$, where the coefficient *α* (GHz/pixel) is measured from the calibration spectrum of water (shift of 7.46 GHz at room temperature) and FSR=30 GHz, given by the VIPAs. Upon completion, the script saves the fitted spectral parameters, i.e. frequency shift (fShift), full-width-at-half-maximum (FWHM) width (fWidth), and amplitude (countsAmplitude), in a location specified by the user, which allows to plot two-dimensional, spatial images of either frequency shift υ_B_ (Fig. 2B), spectral width Γ_B_ (Fig. 2C) or signal amplitude A_B_ (Fig. 2D). We note that no deconvolution to account for spectral broadening has been performed on this dataset and that the dataset and images shown in Fig. 2 was actually composed of two adjacent scan regions (each 200 × 140 µm), which have been stitched together using the Fiji plug-in “Stiching” [5], and Gaussian filtered (width 0.5 pixels). Visco-elastic properties of the sample can then in principle be inferred from the complex longitudinal modulus, which depends on υ_B_ and Γ_B_, as well as on the refractive index and density of the material [6].

## References

[1] Martinez-Morales J.-R., Cavodeassi F., Bovolenta P. (2017). Coordinated morphogenetic mechanisms shape the vertebrate eye. Front. Neurosci..

[2] Scarcelli G., Yun S.H. (2011). Multistage VIPA etalons for high-extinction parallel Brillouin spectroscopy. Opt. Express.

[3] Bevilacqua C., Sánchez-Iranzo H., Richter D., Diz-Muñoz A., Prevedel R. (2019). Imaging mechanical properties of sub-micron ECM in live zebrafish using Brillouin microscopy. Biomed. Opt. Express.

[4] Westerfield M. (2007). The Zebrafish Book – a Guide for the Laboratory use of Zebrafish (Danio Rerio). https://zfin.org/zf_info/zfbook/zfbk.html.

[5] Preibisch S., Saalfeld S., Tomancak P. (2009). Globally optimal stitching of tiled 3D microscopic image acquisitions. Bioinformatics.

[6] Prevedel R., Diz-Muñoz A., Ruocco G., Antonacci G. (2019). Brillouin microscopy: an emerging tool for mechanobiology. Nat. Methods.

